# H_2_O_2_ down-regulates SIRT7’s protective role of endothelial premature dysfunction via microRNA-335-5p

**DOI:** 10.1042/BSR20211775

**Published:** 2022-05-04

**Authors:** Yixin Liu, Jinyu Yang, Xi Yang, Peng Lai, Yi Mou, Juelin Deng, Xinyi Li, Hui Wang, Xiaolei Liu, Lixing Zhou, Linghui Deng, Ziqi Xu, Chun Xiao, Birong Dong

**Affiliations:** 1 National Clinical Research Center for Geriatrics, West China Hospital of Sichuan University, Chengdu, Sichuan Province 610041, China; 2 Department of Geriatrics, West China Hospital of Sichuan University, Chengdu 610041, China; 3 Geriatric Health Care and Medical Research Center, Sichuan University, Chengdu, Sichuan Province 610065, China; 4 School of Food and Bioengineering, Xihua University, Chengdu, China; 5 Geroscience and Chronic Disease Department, The 8th Municipal Hospital for the People, Chengdu, China; 6 Cardiology Department, Hainan Branch of PLA General Hospital, Sanya, China; 7 Medical Examination Center, Aviation Industry Corporation of China 363 Hospital, Chengdu, China; 8 West China School of Basic Medical Sciences and Forensic Medicine, Sichuan University, Chengdu, China

**Keywords:** aging, atherosclerosis, endothelial dysfunction, miR335-5p, SIRT7

## Abstract

Endothelial senescence is believed to constitute the initial pathogenesis of the atherosclerotic cardiovascular disease (ASCVD). MicroRNA-335-5p (miR-335-5p) expression is significantly up-regulated in oxidative stress-induced endothelial cells (ECs). Sirtuin7 (SIRT7) is considered to prevent EC senescence, yet data on its response to ASCVD risk factors are limited. The present study analyzed the elevated levels of miR-335-5p and the decreased levels of SIRT7 in human umbilical vein endothelial cells (HUVECs), and found that high glucose, tumor necrosis factor-α (TNF-α), and H_2_O_2_ are the three contributing factors that induced cellular senescence. The current study also assessed premature endothelial senescence and decreased proliferation, adhesion, migration, and nitric oxide (NO) secretion in HUVECs with these risk factors together with SIRT7–siRNA transfection. It found that the miR-335-5p inhibitor attenuated the down-regulation of SIRT7 expression induced by oxidative stress in HUVECs, and SIRT7 overexpression exerts a rescue effect against miR-335-5p-induced endothelial dysfunction. Furthermore, the direct binding of miR-335-5p to SIRT7 was observed in human embryonic kidney cells 293T (HEK 293T). Therefore, it can be inferred that miR-335-5p down-regulates the expression of SIRT7 in human cells. Current findings may provide deeper insights into the underlying mechanisms of endothelial senescence and potential therapeutic targets of ASCVD as well as other age-related diseases.

## Introduction

Atherosclerotic cardiovascular disease (ASCVD), which involves stroke, myocardial infarction and sudden cardiac death, is currently one of the leading causes of global morbidity and mortality [1]. As expected, the prevalence of ASCVD increases with age, age-related vascular endothelial dysfunction reduces cellular anti-coagulative, anti-proliferative, anti-inflammatory and vasodilatory processes, thereby leading to an increased risk of ASCVD [2]. In fact, the functions of endothelial cells (ECs) are essential to endothelial barrier function, as well-performed proliferation can maintain vascular homeostasis [3], functional adhesion molecules can support and control endothelial junction integrity of vascular lumen [4], and endothelial migration is also the main mechanism of angiogenesis and vascular self-repair [5]. A decrease in endothelial nitric oxide (NO), a significant signaling molecule responsible for maintaining vascular wall tension, results in increased serotonin, enhanced thrombus formation, and vascular contractions [6]. Moreover, senescence-associated β-galactosidase (SA-β-gal), which reflects the increase in lysosomal activity, is the most widely used biomarker for senescent and aging ECs [7]. Endothelial dysfunction is considered as the cause of the initial pathogenesis of atherosclerosis, thereby necessitating a better understanding of EC pathophysiology [3].

Accumulation of cellular damage caused by a shift in the balance between reactive oxygen species (ROS) and antioxidants in favor of oxidants, such as that induced by hyperglycemia and inflammatory factors, is termed oxidative stress [8]. Sustained high ROS levels can contribute to cellular macromolecules damage, endothelial senescence, and hence cause senescence through the activation of inflammatory responses, in turn, leading to the development of atherosclerosis [9]. Previous study found that type 2 diabetes mellitus patients have higher cardiovascular morbidity and mortality compared with non-diabetic patients [10], then glucose-lowering treatment has been proven to be effective in preventing vascular events [11], meanwhile animal studies verified that hyperglycemia can induce vascular cell senescence [12]. The involvement of tumor necrosis factor-α (TNF-α) in the pathogenesis of ASCVD is also established [13]. Numerous *in vitro* studies have also shown that H_2_O_2_, TNF-α, and high glucose treatment could imply a stress-induced senescence phenotype and endothelial dysfunction [12,14–16].

MicroRNAs (miRNAs) are small non-coding RNA molecules that modulate the stability and/or translational efficiency of their target messenger RNAs (mRNAs) by loading an Argonaute protein within the RNA-induced silencing complex (RISC) [17]. The RISC subsequently represses the expression of target mRNAs by either inhibiting their translation or promoting their destabilization. Interestingly, miR-335 expression is significantly up-regulated in aging renal tissues, while microRNA-335-5p (miR-335-5p) is significantly up-regulated in senescent vascular ECs, compared with that in young tissues [18,19].

Sirtuins (e.g., sir2 in yeast or SIRT1 in mammals) are a family of nicotinamide adenine dinucleotide (NAD^+^)-dependent protein deacetylases, which are evolutionarily conserved from bacteria to humans. This protein family is best known for its role in aging and has been shown to prolong the mean and maximal lifespan in many species by promoting genome homeostasis in response to cellular stress. Since the past decade SIRT1 and SIRT6, two of the three mammalian nuclear sirtuins, have been the focus of research related to their protective role as stress adaptors and epigenetic enzymes against inflammation, vascular aging, heart disease, and the development of atherosclerotic plaque [20]. A decline in SIRT7, the third nuclear sirtuins, precedes the manifestations of dangerous DNA lesions, cell death, acute senescence, and shorter lifespan [21,22]. Although signaling events involved in the regulation of SIRT7 expression in ECs exposed to oxidative stress remain poorly understood, it is reasonable to hypothesize that the third member, SIRT7, contributes to maintaining EC functions and preventing EC senescence.

There are limited published data on the regulation of SIRT7 in atherosclerotic endothelial lesions. Based on the previous findings and the information provided by miRTarBase (Supplementary Table S1), a resource for experimentally validated miRNA–target interactions, we hypothesize that miR-335-5p might be involved in promoting the endothelial senescence and modulating the expression of SIRT7, thus exacerbating the dysfunction of ECs. The results obtained in the present study might unravel the mechanisms underlying endothelial senescence and help identify the potential therapeutic targets of atherosclerosis and other age-related diseases.

## Materials and methods

### Cell culture

Human umbilical vein endothelial cells (HUVECs; Medical Discovery Leader, Beijing, China) were cultured in Ham’s F-12K medium (21127022, Gibco, NY, U.S.A.) supplemented with 10% fetal bovine serum (FBS; 16000-044, Gibco, CA, U.S.A.), 1% Penicillin–Streptomycin (1670249, Wegenebio, Shanghai, China) at 37°C in the presence of 5% CO_2_ and saturated humidity. Human embryonic kidney cells 293T (HEK 293T; 4201PAT-CCTCC01526, Cell Bank of Shanghai Chinese Academy of Sciences, Shanghai, China) were cultured in Dulbecco’s modified Eagle’s medium (12634010, Gibco, NY, U.S.A.) supplemented with 10% FBS, 1% Penicillin–Streptomycin at 37°C in the presence of 5% CO_2_ and saturated humidity.

### siRNA and miRNA transfection in HUVECs

The RNA sequences were customized (GenePharma, Shanghai, China). HUVECs (2 × 10^5^/well cells in each chamber) were seeded on to 24-well plates and then transfected with SIRT7–siRNA, an miR-335-5p mimic, an miR-335-5p inhibitor, or a negative control sequence using Lipofectamine 2000 (11668030, Invitrogen, CA, U.S.A.) according to the manufacturer’s protocol. All RNA sequences used in siRNA and miRNA transfections are shown in Supplementary Table S2.

### Grouping and treatment with high glucose, TNF-α, and H_2_O_2_ after SIRT7–siRNA transfection

Forty-eight hours after transfection, HUVECs treated with SIRT7–siRNA or its negative control sequence in the logarithmic growth phase were cultured at a density of 20000 cells/chamber in 24-well plates and divided into four groups: (i) Control: cultured in 10% FBS and DMEM; (ii) H_2_O_2_: cultured in 10% FBS, DMEM, and 60 μmol/l H_2_O_2_ (106058, Tongguang, Beijing, China); (iii) TNF-α: cultured in 10% FBS, DMEM, and 5 ng/ml TNF-α (P02740, Solarbio, Beijing, China); and (iv) high-glucose: cultured in 10% FBS, DMEM, and 50 mmol/l glucose (50-99-7, Xilong Scientific, Guangdong, China). The optimal concentration and time of culture with H_2_O_2_, TNF-α, and glucose were based on previous studies as well as our preliminary experiments (Supplementary Figure S1), which also suggested that these three factors regulate SIRT7 expression in a time- and concentration-dependent manner.

### Grouping and treatment with oxidative stress after transfection with miR-335-5p mimic and inhibitor

Forty-eight hours after transfection using a miR-335-5p mimic and miR-335-5p inhibitor, the HUVECs were cultured and divided into the following six groups, (i) blank: without treatment; (ii) control: treated with transfection agent; (iii) mir-335-5p mimic: transfected with miR-335-5p mimic; (iv) H_2_O_2_: treated with H_2_O_2_ and a transfection agent; (v) H_2_O_2_+mir-335-5p mimic: treated with H_2_O_2_ and transfected with an miR-335-5p mimic; and (vi) H_2_O_2_+mir-335-5p inhibitor: treated with H_2_O_2_ and transfected with an miR-335-5p inhibitor. The transfection of all the groups was performed for 48 h; the groups treated with H_2_O_2_ were cultured in a 60-μmol/l H_2_O_2_ environment for another 48 h.

### Grouping and treatment with oxidative stress after forced SIRT7 and miR-335-5p expression

HUVECs in the logarithmic growth phase were cultured at a density of 20000 cells/chamber in 24-well plates and divided into three groups, (i) control: treated with a transfection agent; (ii) forced SIRT7 expression: transfected with an SIRT7 vector; and (iii) forced SIRT7 and miR-335-5p expression: transfected with SIRT7 and miR-335-5p mimic vectors. After 48 h of transfection, all the groups were cultured in a 60 μmol/l H_2_O_2_ environment for another 48 h. The SIRT7 lentiviral vector with cytomegalovirus promoter (pCDH-CMV-MCS-EF1-CopGFP-T2A-Puro-SIRT7; details are presented in Supplementary Figure S2) and miR-335-5p mimic vectors were customized from Maijin (Beijing, China) and sequenced by GenePharma (Shanghai, China).

### RNA expression analysis

Total RNAs were prepared using TRIzol (RN0102, Aidlab, Beijing, China). RNA samples were reverse transcribed using the HiScript reverse transcriptase kit (R101, Vazyme, Jiangsu, China). The miRNA expression was determined using the qPCR SYBR Green Master Mix (Q111, Vazyme, Jiangsu, China), the primer (sequence information is present in Supplementary Table S2) and QuantStudio 6 Flex Real-Time PCR system (7900 Fast, Applied Biosystems, CA, U.S.A.) according to the manufacturer’s protocol.

### SA-β-gal staining

The SA-β-gal activity was measured using a senescence detection kit (C0602, Beyotime, Shanghai, China) according to the manufacturer’s protocol. Briefly, HUVECs on the plate were exposed to different experimental conditions. The cells were washed thrice with phosphate-buffered saline (10010-049, Life Technologies, Scotland, U.K.), fixed in 2% paraformaldehyde for 3 min at 2°C, washed, and incubated for 16 h at 37°C (without CO_2_) with fresh SA-β-gal stain solution. Then, the cells were photographed. The degree of aging of HUVECs was determined by the intensity of SA-β-gal staining.

### Evaluation of cell proliferation using the cell counting kit-8 assay

After grouping and treatment with ASCVD factors and SIRT7–siRNA transfection, HUVECs (2 × 10^2^/chamber) were seeded on to 96-well plates. Ninety-six hours after transfection, 10 µl of cell counting kit-8 (CCK8; C0037, Beyotime, Shanghai, China) was added to each well and incubated for 4 h at 37°C. The absorbance in each well was measured at 450 nm using a microplate reader.

### Scratch wound healing assay

Measurement of cell migration required the HUVECs (1 ml DMEM containing 10% FBS) to be added to six-well plates at a density of 5 × 10^5^ cells/ml, three wells for each group. Once the cell growth covered 90% of the well, ‘parallel wound gaps’ (each, 0.5 cm) were created between the cell monolayers by scratching with the lower end of the pipette tip. The cells were photographed after three washes with PBS and incubated for 48 h. The area of the wounds measured by ImageJ, an open-source software.

### Cell adhesion assay

Following 48-h transfection, the HUVECs’ suspensions (1 × 10^4^/ml) were added to 96-well plates coated with serum-free Ham’s F-12K medium (21127022, Gibco, NY, U.S.A.) and 100 μg/ml Matrigel basement membrane matrix (356234, BD, Biosciences, MA, U.S.A.) collagen. The separated cells were washed with PBS and fixed with 5% glutaraldehyde at 4°C for 30 min. The cells were then incubated at 37°C in the presence of 5% CO_2_. After 1 h, the cells that adhered to the substrate were stained with 0.1% Crystal Violet (G1064, Solarbio, Beijing, China) for 30 min. Next, the stained cells were solubilized with 100 μl of 10% acetic acid. Finally, the absorbance of the solution was measured at 595 nm.

### NO assay

NO secretion was detected using the NO assay kit (A012, Jiancheng Bioengineering, Jiangsu, China). An aliquot of medium 100 μl from each culture well was mixed with 200 μl of nitrate reductase for the conversion of nitrate into nitrite, followed by 100 μl of the Griess reagent. The mixture was incubated for 10 min at 25 ± 2°C to allow the color to develop, and the absorbance at 550 nm was measured in a microplate reader (Nano-100, Aosheng Devices, Zhejiang, China).

### Western blot analysis

The supernatant fluid of HUVECs culture was applied to a 10% polyacrylamide gel and electrophoresed at 60–90 V to investigate the components of SIRT7. The SIRT7 proteins were then transferred to nitrocellulose membranes. The membranes were blocked with nonfat dry milk for 2 h and incubated overnight with anti-SIRT7 antibodies (DF6161, Affinity Biosciences, Jiangsu, China). HRP-conjugated goat anti-rabbit IgG (BA1039, Boster Biological Technology, Hubei, China) was used as the secondary antibody. The membranes were briefly incubated with ECL detection reagent (ECL-00114, Dingguo, Beijing, China). The protein was visualized and analyzed using the Fusion FX7 system (Peqlab, Erlangen, Germany). Anti-GAPDH antibodies (AB-P-R001,Goodhere Biotechnology, Zhejiang, China) was used as an internal control. All raw Western blot data of this article are presented in Supplementary Figure S3.

### Plasmid construction and luciferase assay

The miRNA target prediction online database (miRTarBase) was used to predict the binding sites of miR-335-5p on SIRT7 (as shown in Supplementary Table S1). The 3′-UTR of SIRT7 was synthesized and sequenced by GenePharma (Shanghai, China). The PCR products were subcloned into the region directly downstream of the stop codon in the luciferase gene in the luciferase reporter vector to generate a p-Luc-UTR reporter plasmid (as shown in Supplementary Table S1 and Figure S4). Overlap PCR was used to construct a 3′-UTR mutant reporter plasmid. The primers used to generate SIRT7 3′-UTR were miR-335-5p mimic (UCAAGAGCAAUAACGAAAAAUGU) and mimic control (UUCUCCGAACGUGUCACGUTT) (GenePharma, Shanghai, China). The sequences used for mutant 3′-UTR were confirmed by sequencing.

HEK 293 cell line exhibit a higher growth rate, sensitivity, accuracy, stability and effectiveness in luciferase assay compared with HUVECs [23,24]. The luciferase assay required the HEK 293T cells to be seeded in six-well plates and co-transfected with a mixture of the miRNA mimic or the mimic control pYr-KL 3′-UTR, and *Renilla* luciferase vector pRL-CMV (E2261, Promega, WI, U.S.A.) using the Lipofectamine 2000 (11668030, Invitrogen, CA, U.S.A.) transfection system. The Firefly and *Renilla* luciferase activities in the cell lysates were measured using the dual-luciferase reporter assay system (E1910, Promega, WI, U.S.A.) 48 h after transfection.

### Statistical analysis

Data entry, statistical analyses, and presentation were performed using SPSS version 21.0 (SPSS, IL, U.S.A.) and GraphPad Prism 8.0.1 software (GraphPad Software Inc., CA, U.S.A.). Measurement data are summarized as mean ± standard deviation. A normality test was realized when the number of samples was sufficient (Shapiro–Wilk test). If samples followed a Gaussian distribution, one-way analysis of variance (ANOVA) was used to compare the differences in outcomes between groups. When samples did not pass the normality test, a non-parametric test (Mann–Whitney test) was applied. A *P*-value of less than 0.05 was used as a cutoff to define statistical significance.

## Results

### H_2_O_2_, TNF-α, and high glucose down-regulate SIRT7 and up-regulate miR-335-5p expression in ECs

**Figure 1:**
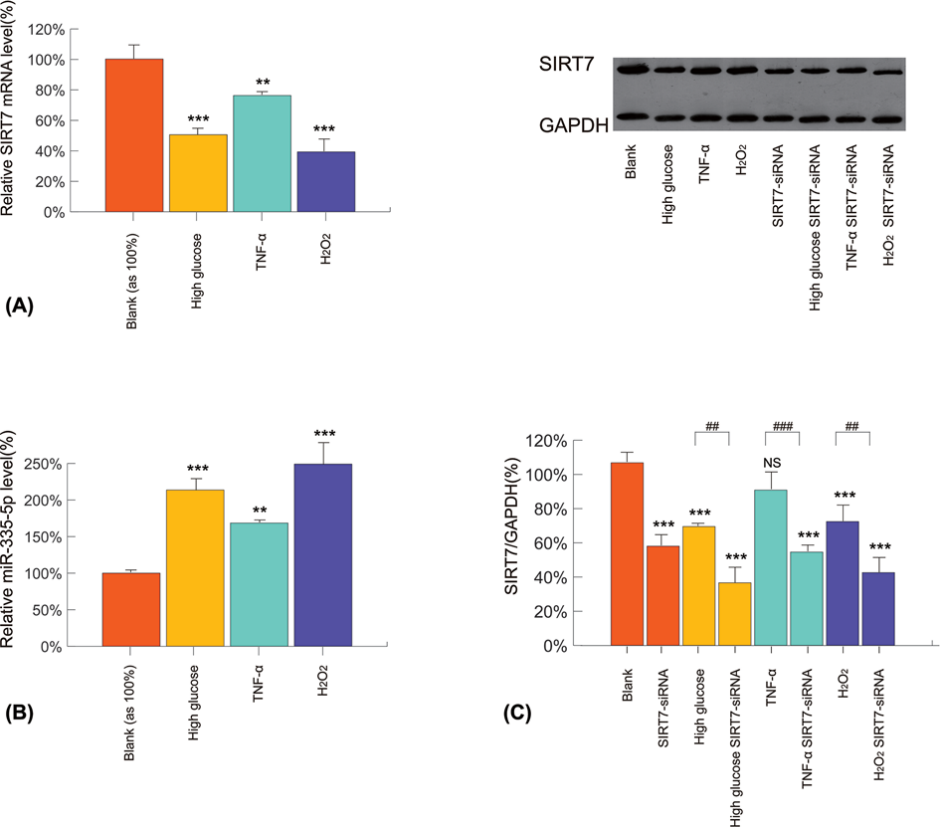
miR-335-5p and SIRT7 expression following treatment with high glucose, TNF-α, and H_2_O_2_ (**A**) Relative miR-335-5p expression in HUVECs determined using RT-PCR assay (*n*=3, in all groups). (**B**) Relative SIRT7 expression in HUVECs determined using RT-PCR assay (*n*=3, in all groups). (**C**) Western blot assay on HUVECs for SIRT7 expression following SIRT7–siRNA transfection (*n*=3, in all groups). NS: non-significance, **P<0.01 versus the blank or control group, ***P<0.005 versus the blank or control group, ^##^*P*<0.01 versus the underlined group, ^###^*P*<0.005 versus the underlined group. All raw Western blots data of this article are presented in Supplementary Figure S4. Values of all groups followed a Gaussian distribution.

Real-time PCR was used to assess SIRT7 mRNA transcription and miR-335-5p expression levels in the three experimental groups. SIRT7 mRNA levels in these three experimental groups were found to be significantly lower in the high-glucose, TNF-α, and H_2_O_2_-induced groups, respectively (50.4 ± 4.0, 76.1 ± 2.0, and 39.2 ± 8.0%) than those in the negative control, while the miR-335-5p levels were found to be higher (213.7 ± 15.7, 168.3 ± 3.1, and 249.0 ± 30.0%) than those in the negative control (Figure 1A,B). Western blot analysis also suggested expression of SIRT7 protein was down-regulated (65.0 ± 1.1, 85.0 ± 7.6, and 67.8 ± 7.2%, shown in Figure 1C).

### SIRT7 silencing aggravates oxidative damage, inflammation, and high-glucose-induced premature senescence in ECs

The quantitative real-time PCR results showed that transfection with SIRT7–siRNA suppressed the expression of SIRT7 mRNA by more than sevenfold (Supplementary Figure S5), and Western blot analysis results indicated similar suppression at the protein level (Figure 1C).

**Figure 2:**
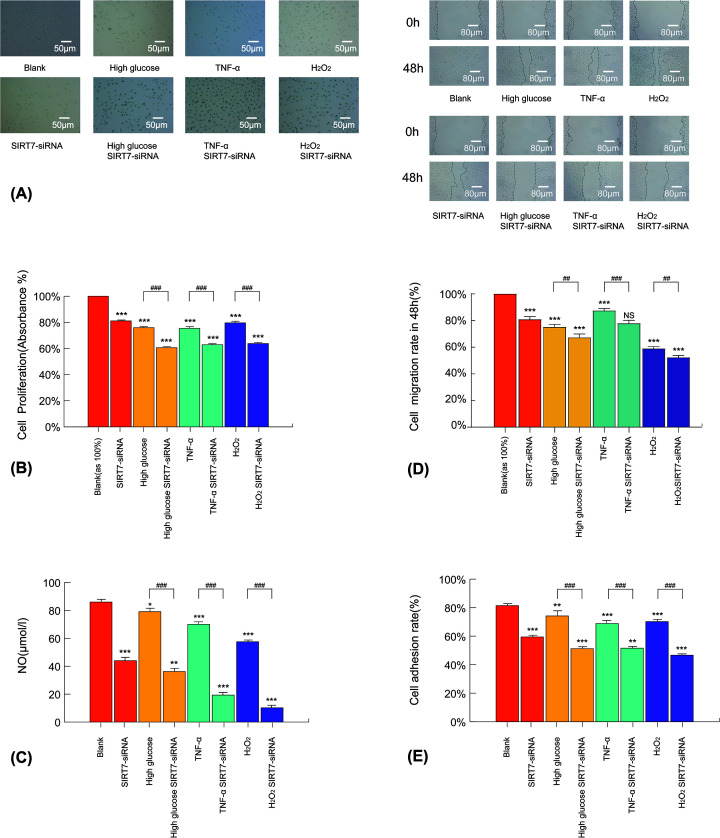
SIRT7 silencing aggravates oxidative damage, inflammation, and high-glucose-induced dysfunction in HUVECs (**A**) SA-β-gal staining in different experimental groups 48 h after treatments (*n*=3, in all groups). (**B**) Proliferation in different experimental groups (*n*=3, in all groups). (**C**) NO secretion of HUVECs in different experimental groups (*n*=3, in all groups). (**D**) Migration in different experimental groups (*n*=3, in all groups). (**E**) Adhesion in different experimental groups (*n*=3, in all groups; high-glucose SIRT7–siRNA, TNF-α SIRT7–siRNA, and H_2_O_2_ SIRT7–siRNA groups were compared with the group only treated with SIRT7–siRNA). *P<0.05 versus the blank or control group, **P<0.01 versus the blank or control group, ***P<0.005 versus the blank or control group, ^##^*P*<0.01 versus the underlined group, ^###^*P*<0.005 versus the underlined group. Values of all groups followed a Gaussian distribution.

H_2_O_2_, TNF-α, and high-glucose stimulation increased SA-β-gal staining of ECs, which was aggravated further after SIRT7 down-regulation. These findings indicated that SIRT7 might attenuate the phenotypic changes resulting in EC aging in the presence of H_2_O_2_, TNF-α, and high glucose (Figure 2A). The CCK8 cell proliferation assay analysis showed that the proliferation of HUVECs was lower under H_2_O_2_, TNF-α, and high glucose exposure compared with that of the control groups. SIRT7 suppression was shown to significantly decrease the proliferation of HUVECs in all the groups (Figure 2B). The migration assay showed that SIRT7–siRNA transfection down-regulated the migration of HUVECs with or without oxidative stress, inflammatory factors, and high glucose levels (Figure 2C). Consistent with previous findings, the adhesion rate and NO secretion of HUVECs significantly decreased after transfection with SIRT7–siRNA with or without H_2_O_2_, TNF-α, and high-glucose treatments (Figure 2D,E). Generally, H_2_O_2_ has a stronger down-regulation effect than TNF-α and high glucose on NO secretion, proliferation, migration, and adhesion with SIRT7–siRNA (Supplementary Figure S6).

### SIRT7 overexpression rescue endothelial function from oxidative stress and miR-335-5p forced expression attenuate the rescue effect

**Figure 3:**
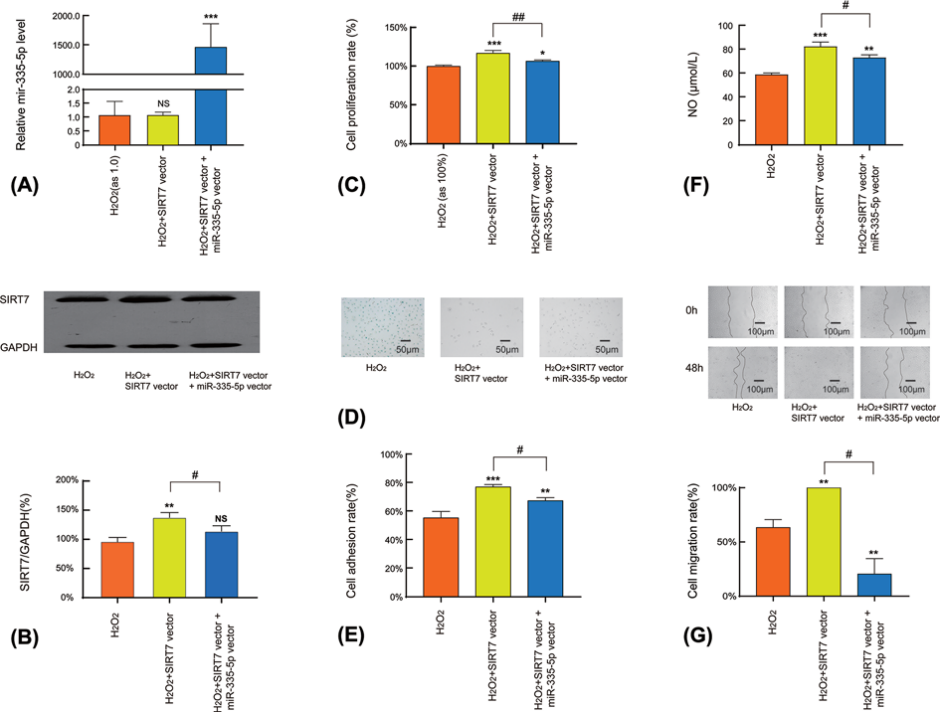
Rescue effect of SIRT7 in oxidative stress-induced endothelial senescence in HUVECs (**A**) Relative miR-335-5p level following forced SIRT7 and miR-335-5p overexpression (*n*=3, in all groups). (**B**) Western blot assay for SIRT7 in different experimental groups (*n*=3, in all groups). (**C**) Proliferation in different experimental groups (*n*=3, in all groups). (**D**) SA-β-gal staining in different experimental groups (*n*=3, in all groups). (**E**) Adhesion in different experimental groups (*n*=3, in all groups). (**F**) NO secretion in different experimental groups (*n*=3, in all groups). (**G**) Migration in different experimental groups (*n*=3, in all groups). *P<0.05 versus the blank or control group, **P<0.01 versus the blank or control group, ***P<0.005 versus the blank or control group, ^#^*P*<0.05 versus the underlined group, ^##^*P*<0.01 versus the underlined group. Values of all groups followed a Gaussian distribution.

RT-PCR analysis of HUVECs indicated that forced *SIRT7* overexpression did not relatively affect miR-335-5p levels (Figure 3A). The Western blot analysis of HUVECs revealed that SIRT7 expression was significantly elevated after *SIRT7* transfection; however, this elevation was not significant in the cells co-transfected with miR-335-5p (Figure 3B). The CCK8 cell proliferation assay analysis indicated that the H_2_O_2_-induced down-regulation of the proliferation of HUVECs was rescued by transfection with *SIRT7*; the co-transfection of miR-335-5p limited the rescue effect (Figure 3C). Migration, adhesion, NO secretion, and SA-β-gal staining assay results of the groups transfected with SIRT7 and co-transfected with miR-335-5p indicated similar proliferation assay results (Figure 3D–G). These findings indicate that transfection with *SIRT7* exerts a rescue effect against endothelial dysfunction and immature senescence induced by treatment with H_2_O_2_; however, the rescue effect was partly suppressed by miR-335-5p, which acts as an upstream factor in the SIRT7 pathway.

### miR-335-5p regulates SIRT7 expression under oxidative stress and targets SIRT7 3′-UTR

**Figure 4:**
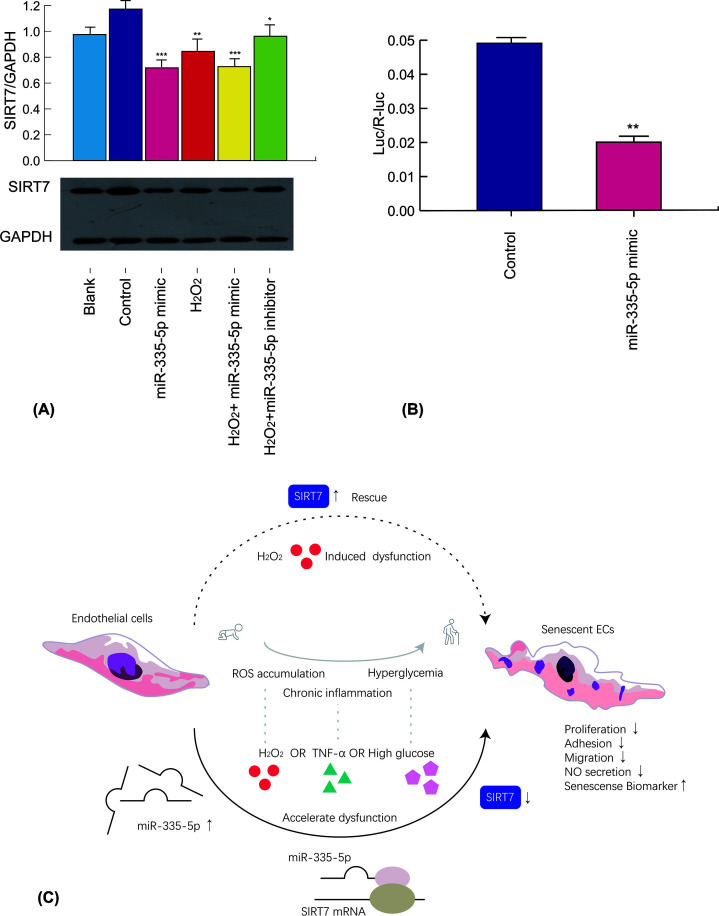
miR-335-5p regulates SIRT7 expression under oxidative stress and targets SIRT7 3′-UTR (**A**) SIRT7 expression following miR-335-5p mimic and inhibitor transfection and H_2_O_2_ treatment in HUVECs (*n*=3, in all groups). (**B**) SIRT7 3′-UTR dual-luciferase assay in HEK 293T (*n*=3, in both groups). (**C**) Visualized summary. *P<0.05 versus the blank or control group, **P<0.01 versus the blank or control group, ***P<0.005 versus the blank or control group. Values of all groups followed a Gaussian distribution.

Western blot analysis showed that the overexpression of miR-335-5p decreased SIRT7 expression in HUVECs (miR-335-5p mimic: 64.3 ± 3.8% vs. control: 131.0 ± 6.0%; *P*<0.01). The analysis also showed that the miR-335-5p inhibition partly attenuated the reduction in H_2_O_2_-induced SIRT7 expression (inhibition + H_2_O_2_: 79.7 ± 3.2% vs. mimic+H_2_O_2_: 55.3 ± 4.2%; *P*<0.01) (Figure 4A). A dual-luciferase assay was performed to confirm the direct binding of SIRT7 to miR-335-5p. HEK 293T was transfected with a luciferase reporter plasmid along with an miR-335-5p mimic or negative control. Notably, the ratio of Luc/R-luc was significantly lower in the SIRT7 3′-UTR + miR-335-5p mimic group than in the SIRT7 3′-UTR + control group (miR-335-5p 0.0199 ± 0.00139 vs. control 0.0489 ± 0.00168; *P*<0.01). The results, therefore, suggested that miR-335-5p regulated SIRT7 by directly binding to the SIRT7 3′-UTR (Figure 4B).

## Discussion

The present study demonstrated that H_2_O_2_ (ROS accumulation model in the elderly), TNF-α (chronic low dosage inflammation model in the elderly), and high glucose (hyperglycemia model as observed in diabetes) induced HUVECs senescence, up-regulated the expression of miR-335-5p, and down-regulated the expression of SIRT7. It also demonstrated a novel role of SIRT7, the inhibition of which led to premature endothelial senescence; maintenance of proliferation, adhesion, migration, and NO secretion in the endothelium; and intolerance to H_2_O_2_, TNF-α, and high-glucose treatment in ECs. Notably, the overexpression of miR-335-5p decreased the expression of SIRT7 in ECs, and miR-335-5p inhibitor attenuated the down-regulation of SIRT7 expression, which was induced by oxidative stress. Furthermore, SIRT7 overexpression rescued endothelial function from oxidative stress, and miR-335-5p forced expression attenuate the rescue effect. Thus, the current study revealed that miR-335-5p targeted and bound to *SIRT7* mRNA in human cells (Figure 4C).

Previous findings together with the current results enable a detailed understanding of the multiple roles that miR-335-5p plays in age-related diseases and process of cancerous lesion formation through different regulatory networks. MiR-335-5p might behave as a regulator with oxidative stress and cellular lesions, as well as an endothelial ROS-induced signal of the senescence-associated secretory phenotype (SASP), which contributes to atherosclerosis. In our earlier study, it showed that forced endothelial miR-335-5p expression aggravates premature endothelial senescence and dysfunction via a decrease in the expression of *sKlotho*, the first anti-aging gene to have been discovered [25,26]. During senescence, miR-335-5p levels are significantly increased in renal residential cells and the hippocampus in patients with Alzheimer’s disease and dementia [27]. Meanwhile, miRNA-335-5p could suppress the proliferation and invasion of epithelial cancer cells and acute inflammatory responses. Along with the results of previous studies, the current results on miR-335-5p may benefit further studies on cardiovascular disease focused on the deeper relationship among the accumulation of oxide stress, adaptation of cellular senescence, and phenotype of aging or atherosclerosis. Additionally, the results may facilitate the use of miR-335-5p as a potential SASP inhibitor target or a prospective biomarker for EC response to oxide stress.

The intrinsic mechanism underlying the protection exerted by SIRT7 on an individual from premature endothelial senescence and atherosclerosis is yet to be explored. The majority of the functions of SIRT7 are mediated via enzymatic activities, including the highly selective deacetylation of histone H3K18 and desuccinylation of H3K122. SIRT7 is recruited in a PARP1-dependent manner to the sites of DNA double-strand breaks in senescent cells [28]. Moreover, SIRT7 might involve in telomere maintenance via TR4, regulation of mitochondrial ribosomal proteins via GABP. Accumulating evidence has demonstrated that cellular proliferation and migration are regulated by SIRT7 signaling through the Wnt/β-catenin pathway [29]. Furthermore, SIRT7 prevents cell death, including apoptosis [30]. Briefly, experimental evidence suggests that SIRT7 plays a protective role in vascular endothelial senescence. Based on the current results, future SIRT7 studies might explore the mechanism underlying endothelial homeostasis in age-related diseases, and a treatment strategy for atherosclerosis based on its rescue effect in oxide stress-induced endothelial dysfunction.

The data in the current article strongly support the correlation between miR-335-5p and SIRT7. Our experiments contribute that SIRT7 may perform as a promising target to reduce the risk of ASCVD, in particular, to reduce the development of premature endothelial senescence.

One limitation of the present study is the lack of the data on the effect of SIRT7 and miR-335-5p on animal models of atherosclerosis or endothelial senescence. The potential miR-335-5p-based diagnostic or SIRT7-targeted therapeutic application in age-related diseases still depends on future trials with more specific design and more quantitative results, especially those focusing on the downstream mechanism of SIRT7 improving endothelial dysfunction.

## Supplementary Material

Supplementary Figures S1-S6 and Tables S1-S2

## Data Availability

All data generated or analyzed during the present study are included in this published article, supplementary files and following URL: https://data.mendeley.com/datasets/bwm9z69r66/draft?a=05f72999-ad4d-4de5-aa3d-08acd56784d4.

## Abbreviations

ASCVD: atherosclerotic cardiovascular disease
CCK8: cell counting kit-8
EC: endothelial cell
FBS: fetal bovine serum
HEK 293T: human embryonic kidney cells 293T
HUVEC: human umbilical vein endothelial cell
miRNA: microRNA
miR-335-5p: microRNA-335-5p
mRNA: messenger RNA
NO: nitric oxide
RISC: RNA-induced silencing complex
ROS: reactive oxygen species
SASP: senescence-associated secretory phenotype
SA-β-gal: senescence-associated β-galactosidase
SIRT: Sirtuin
TNF-α: tumor necrosis factor-α

## References

[1] Virani S.S., Alonso A., Aparicio H.J., Benjamin E.J., Bittencourt M.S., Callaway C.W. et al. (2021) Heart Disease and Stroke Statistics-2021 Update: a report from the American Heart Association. Circulation 143, e254–e743 10.1161/CIR.0000000000000950 33501848 PMC13036842

[2] Seals D.R., Kaplon R.E., Gioscia-Ryan R.A. and LaRocca T.J. (2014) You’re only as old as your arteries: translational strategies for preserving vascular endothelial function with aging. Physiology (Bethesda) 29, 250–264 10.1152/physiol.00059.2013 24985329 PMC4103060

[3] Gimbrone M.A. Jr and Garcia-Cardena G. (2016) Endothelial cell dysfunction and the pathobiology of atherosclerosis. Circ. Res. 118, 620–636 10.1161/CIRCRESAHA.115.306301 26892962 PMC4762052

[4] Reglero-Real N., Colom B., Bodkin J.V. and Nourshargh S. (2016) Endothelial cell junctional adhesion molecules: role and regulation of expression in inflammation. Arterioscler. Thromb. Vasc. Biol. 36, 2048–2057 10.1161/ATVBAHA.116.307610 27515379 PMC5035539

[5] Pellet-Many C. (2015) Chemotactic migration of endothelial cells towards VEGF-A(1)(6)(5). Methods Mol. Biol. 1332, 151–157 10.1007/978-1-4939-2917-7_11 26285752

[6] Yang Z. and Ming X.F. (2006) Recent advances in understanding endothelial dysfunction in atherosclerosis. Clin. Med. Res. 4, 53–65 10.3121/cmr.4.1.53 16595793 PMC1435659

[7] Kurz D.J., Decary S., Hong Y. and Erusalimsky J.D. (2000) Senescence-associated (beta)-galactosidase reflects an increase in lysosomal mass during replicative ageing of human endothelial cells. J. Cell Sci. 113, 3613–3622 10.1242/jcs.113.20.3613 11017877

[8] Birben E., Sahiner U.M., Sackesen C., Erzurum S. and Kalayci O. (2012) Oxidative stress and antioxidant defense. World Allergy Organ J. 5, 9–19 10.1097/WOX.0b013e3182439613 23268465 PMC3488923

[9] Nowak W.N., Deng J., Ruan X.Z. and Xu Q. (2017) Reactive oxygen species generation and atherosclerosis. Arterioscler. Thromb. Vasc. Biol. 37, e41–e52 10.1161/ATVBAHA.117.309228 28446473

[10] Gu K., Cowie C.C. and Harris M.I. (1999) Diabetes and decline in heart disease mortality in US adults. JAMA 281, 1291–1297 10.1001/jama.281.14.1291 10208144

[11] Riddle M.C., Gerstein H.C., Holman R.R., Inzucchi S.E., Zinman B., Zoungas S. et al. (2018) A1C targets should be personalized to maximize benefits while limiting risks. Diabetes Care 41, 1121–1124 10.2337/dci18-0018 29784695

[12] Shosha E., Xu Z., Narayanan S.P., Lemtalsi T., Fouda A.Y., Rojas M. et al. (2018) Mechanisms of diabetes-induced endothelial cell senescence: role of arginase 1. Int. J. Mol. Sci. 19, 1–15 10.3390/ijms19041215 29673160 PMC5979610

[13] Meldrum D.R., Cleveland J.C. Jr, Cain B.S., Meng X. and Harken A.H. (1998) Increased myocardial tumor necrosis factor-alpha in a crystalloid-perfused model of cardiac ischemia-reperfusion injury. Ann. Thorac. Surg. 65, 439–443 10.1016/S0003-4975(97)01297-6 9485242

[14] Suo R., Zhao Z.Z., Tang Z.H., Ren Z., Liu X., Liu L.S. et al. (2013) Hydrogen sulfide prevents H(2)O(2)-induced senescence in human umbilical vein endothelial cells through SIRT1 activation. Mol. Med. Rep. 7, 1865–1870 10.3892/mmr.2013.1417 23588928

[15] Khan S.Y., Awad E.M., Oszwald A., Mayr M., Yin X., Waltenberger B. et al. (2017) Premature senescence of endothelial cells upon chronic exposure to TNFalpha can be prevented by N-acetyl cysteine and plumericin. Sci. Rep. 7, 39501 10.1038/srep39501 28045034 PMC5206708

[16] Maeda M., Hayashi T., Mizuno N., Hattori Y. and Kuzuya M. (2015) Intermittent high glucose implements stress-induced senescence in human vascular endothelial cells: role of superoxide production by NADPH oxidase. PLoS ONE 10, e0123169 10.1371/journal.pone.0123169 25879533 PMC4400006

[17] Gurtan A.M. and Sharp P.A. (2013) The role of miRNAs in regulating gene expression networks. J. Mol. Biol. 425, 3582–3600 10.1016/j.jmb.2013.03.007 23500488 PMC3757117

[18] Zhang J.B., Zhu X.N., Cui J., Chen P., Wang S.M. and Wang J.S. (2012) Differential expressions of microRNA between young and senescent endothelial cells. Zhonghua Yi Xue Za Zhi 92, 2205–2209 23158428

[19] Bai X.Y., Ma Y., Ding R., Fu B., Shi S. and Chen X.M. (2011) miR-335 and miR-34a promote renal senescence by suppressing mitochondrial antioxidative enzymes. J. Am. Soc. Nephrol. 22, 1252–1261 10.1681/ASN.2010040367 21719785 PMC3137573

[20] D’Onofrio N., Servillo L. and Balestrieri M.L. (2018) SIRT1 and SIRT6 signaling pathways in cardiovascular disease protection. Antioxid. Redox Signal. 28, 711–732 10.1089/ars.2017.7178 28661724 PMC5824538

[21] Wyman A.E., Noor Z., Fishelevich R., Lockatell V., Shah N.G., Todd N.W. et al. (2017) Sirtuin 7 is decreased in pulmonary fibrosis and regulates the fibrotic phenotype of lung fibroblasts. Am. J. Physiol. Lung Cell. Mol. Physiol. 312, L945–L958 10.1152/ajplung.00473.2016 28385812 PMC5495944

[22] Vazquez B.N., Thackray J.K. and Serrano L. (2017) Sirtuins and DNA damage repair: SIRT7 comes to play. Nucleus 8, 107–115 10.1080/19491034.2016.1264552 28406750 PMC5403131

[23] Sonnenschein K., Fiedler J., Pfanne A., Just A., Mitzka S., Geffers R. et al. (2019) Therapeutic modulation of RNA-binding protein Rbm38 facilitates re-endothelialization after arterial injury. Cardiovasc. Res. 115, 1804–1810 10.1093/cvr/cvz063 30843048 PMC6755352

[24] Wang L., Xu G.L., Gao K., Wilkinson J., Zhang F., Yu L. et al. (2016) Development of a robust reporter-based assay for the bioactivity determination of anti-VEGF therapeutic antibodies. J. Pharm. Biomed. Anal. 125, 212–218 10.1016/j.jpba.2016.03.042 27042807

[25] Liu Y., Lai P., Deng J., Hao Q., Li X., Yang M. et al. (2019) Micro-RNA335-5p targeted inhibition of sKlotho and promoted oxidative stress-mediated aging of endothelial cells. Biomark. Med. 13, 457–466 10.2217/bmm-2018-0430 30785341

[26] Kuro-o M., Matsumura Y., Aizawa H., Kawaguchi H., Suga T., Utsugi T. et al. (1997) Mutation of the mouse klotho gene leads to a syndrome resembling ageing. Nature 390, 45–51 10.1038/36285 9363890

[27] Bottero V. and Potashkin J.A. (2019) Meta-analysis of gene expression changes in the blood of patients with mild cognitive impairment and Alzheimer’s disease dementia. Int. J. Mol. Sci. 20, 1–23 10.3390/ijms20215403 31671574 PMC6862214

[28] Vazquez B.N., Thackray J.K., Simonet N.G., Kane-Goldsmith N., Martinez-Redondo P., Nguyen T. et al. (2016) SIRT7 promotes genome integrity and modulates non-homologous end joining DNA repair. EMBO J. 35, 1488–1503 10.15252/embj.201593499 27225932 PMC4884211

[29] Zheng J., Chen K., Wang H., Chen Z., Xi Y., Yin H. et al. (2018) SIRT7 regulates the vascular smooth muscle cells proliferation and migration via Wnt/beta-catenin signaling pathway. Biomed Res. Int. 2018, 4769596 10.1155/2018/4769596 30627559 PMC6304541

[30] Vakhrusheva O., Smolka C., Gajawada P., Kostin S., Boettger T., Kubin T. et al. (2008) Sirt7 increases stress resistance of cardiomyocytes and prevents apoptosis and inflammatory cardiomyopathy in mice. Circ. Res. 102, 703–710 10.1161/CIRCRESAHA.107.164558 18239138

