# Supplementary material for: H2O2 down-regulates SIRT7’s protective role of endothelial premature dysfunction via microRNA-335-5p
Source: Biosci Rep. 2022 May 4;42(5):BSR20211775. doi: 10.1042/BSR20211775 (PMC9093694; doi:10.1042/BSR20211775)
Supplement: Supplementary Figures S1-S6 and Tables S1-S2 [file BSR-2021-1775_supp.pdf]

Supplementary Material

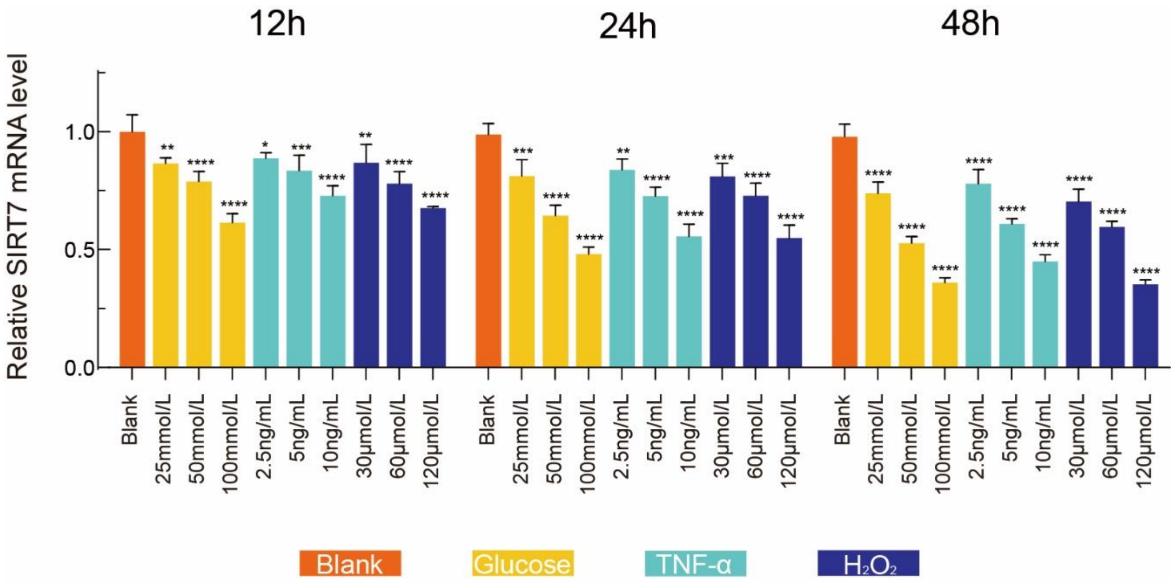

Supplementary Figure 1. Time and concentration manner of ASCVD factors regulated SIRT7 expression.

*P < 0.05 versus blank group, **P < 0.01, ***P < 0.005, ****P < 0.001.

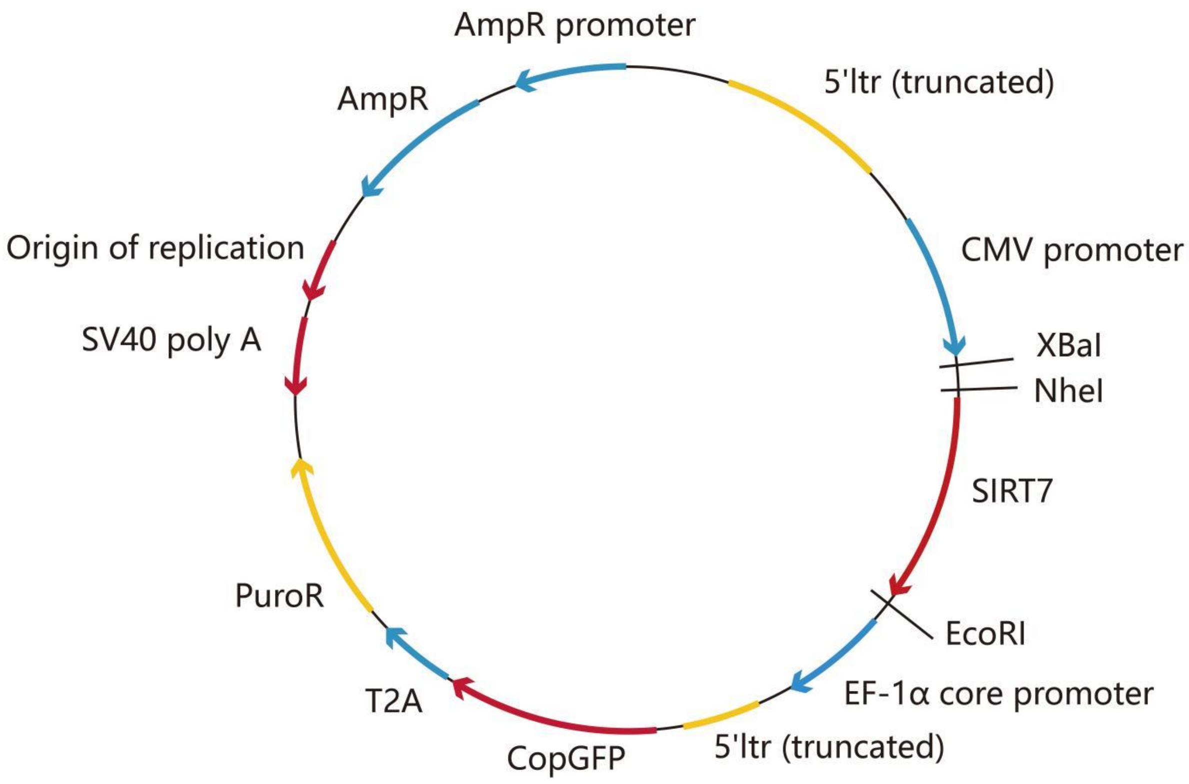

Supplementary Figure 2. Structure of SIRT7 vector.

XBa I, cut site of restriction enzyme isolated from the *Xanthomonas badrii*. Nhe I, cut site of restriction enzyme isolated from *Neisseria mucosa heidelbergensis*. EcorR I, cut site of restriction enzyme isolated from *Escherichia coli*. CMV, Cytomegalovirus. EF-1, Elongation Factor-1. 5'ltr, 5'long terminal repeat. CopGFP, *Copepod* green fluorescent protein. T2A, 2A self-cleaving peptides from *Thosea asigna*. PuroR, Puromycin-resistance gene. AmpR, Ampicillin-resistance gene. SV40, Simian vacuolating virus 40.

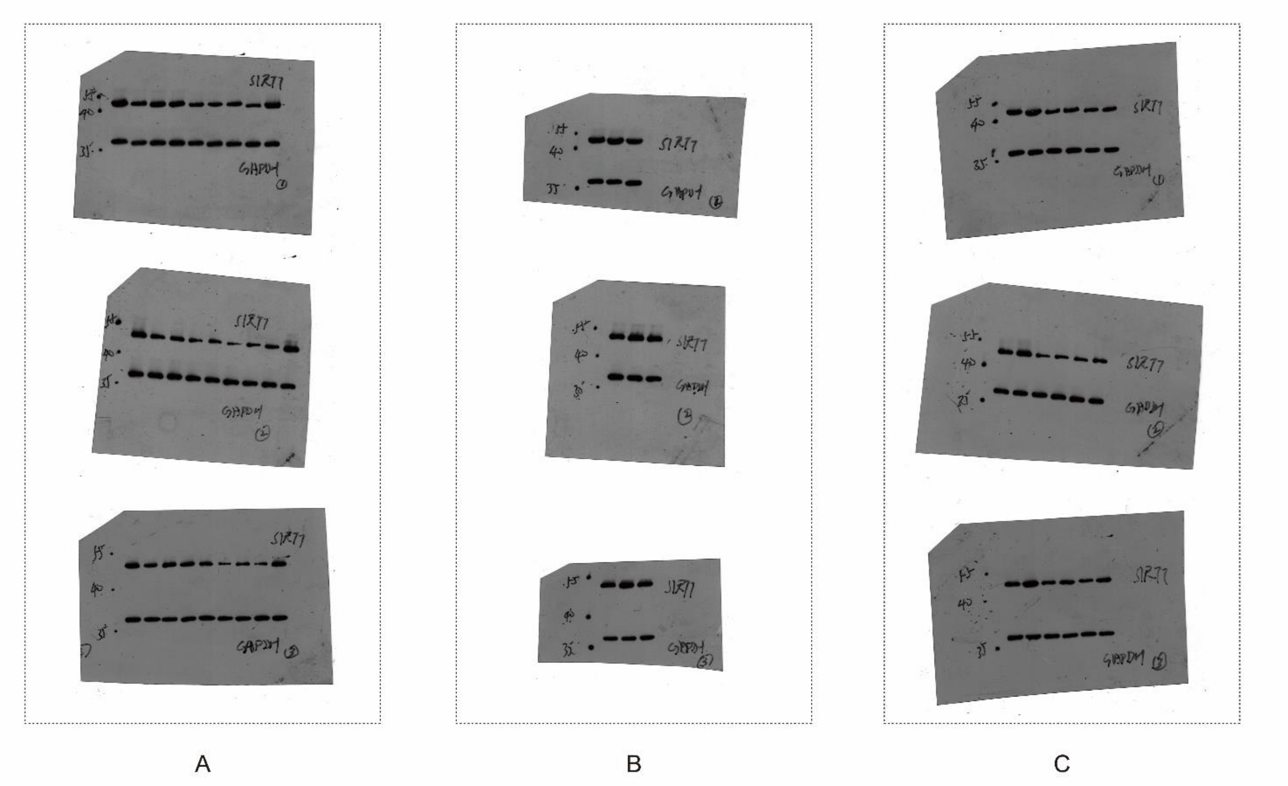

Supplementary Figure 3. Raw western blot in current paper.

(A) WB images of Figures1C (HUVECs western blot assay for SIRT7 expression with a SIRT7-siRNA transfection). **(B)** WB images of Figures3B (Western blot assay for SIRT7 in different experimental groups). **(C)** WB images of Figures4A (SIRT7 expression with mir-335-5p mimic and inhibitor transfection and H_2O_2 treatment in HUVECs). The lanes of **(A)** (from left to right) were blank, High glucose, TNF-α, H_2O_2, SIRT7-siRNA, High glucose SIRT7-siRNA, TNF-α SIRT7-siRNA, H_2O_2 SIRT7-siRNA and blank repeating. The lanes of **(B)** were H_2O_2, H_2O_2+SIRT7-siRNA vector, H_2O_2+SIRT7-siRNA vector+miR-335-5p vector. The lanes of **(C)** were blank, control, miR-335-5p mimic, H_2O_2, H_2O_2+miR-335-5p mimic, H_2O_2+miR-335-5p inhibitor.

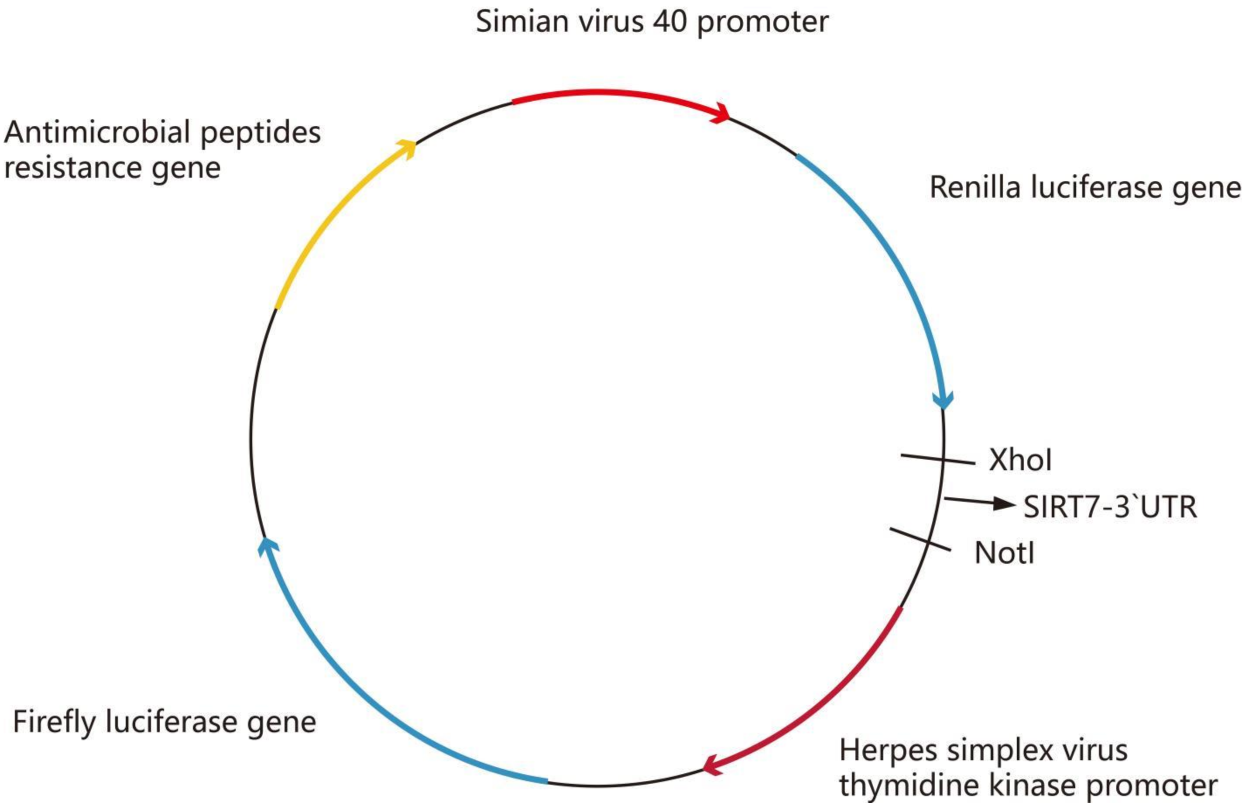

Supplementary Figure 4. Structure of luciferase reporter vector.

XhoI, cut site of restriction enzyme isolated from *Xanthomonas vasicola*. NotI, cut site of restriction enzyme isolated from *Nocardia otitidiscaviarum*.

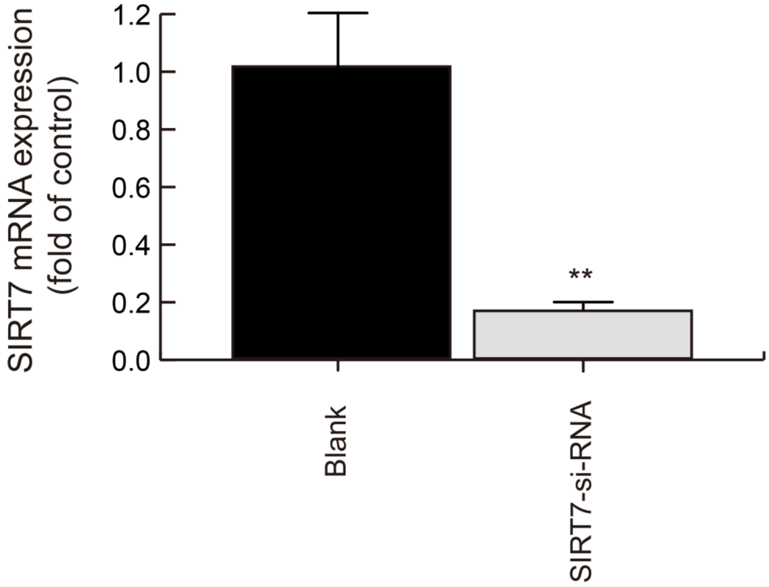

Supplementary Figure 5. SIRT7-siRNA silencing SIRT7 mRNA expression in HUVECs.

Blank: HUVECs transfected with the negative control sequence. SIRT7-siRNA: HUVECs transfected with the SIRT7-siRNA sequence.

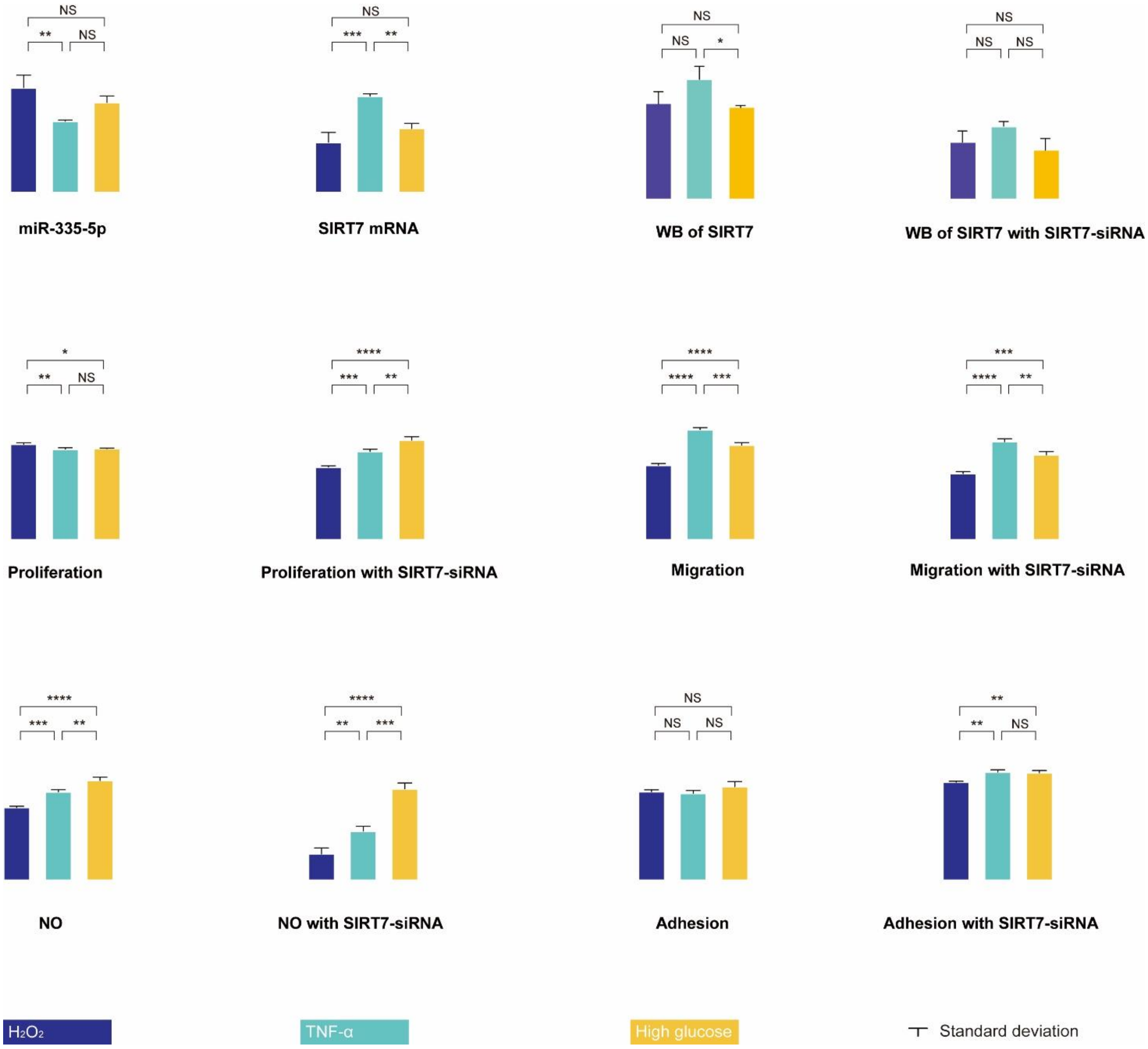

Supplementary Figure 6. Significance among the three risk factors endothelial effect (Expanding of Figures.1A,1B,1C,2B,2C,3B,3C,3E).

NS non significance versus compared group, *P < 0.05 versus compared group, **P < 0.01, ***P < 0.005.

Supplementary Table 1. SIRT7-3'UTR Sequence in the luciferase reporter vector and predicted interaction with miR-335-5p

SIRT7-3'UTR Sequence in the luciferase reporter vector (5'-3')

1
CTCGAGGGGAGAACGAACTCTTTGGGGATGACATTTTCACCGTGACATTTTTAGCCA
TTT

61
GTCCTTGAGGAAGCCCCTTGCACTGCTGCGGTTGTACCCTGATACGGCCTGGCCAT
CGAG

121
GACACCTGCCCATCCGGCCTCTGTGTCAAGAGGTGGCAGCCGCACCTTTCTGTGAG
AACG

181
GAACTCGGGTTATTTCAGCCCCGGCCTGCAGAGTGGAAGCGCCCAGCGGCCTTTCC
TCGC

241
TCACCAGGCCAGTCTCAGGGCCTCACCGTATTTCTACTACTACTTAATGAAAAGTGT
GA

301
ACTTTATAGAATCCTCTCTGTACTGGATGTGCGGCAGAGGGGTGGCTCCGAGCCTC
GGCT

361 CTATGCAGACCTTTTTATTTCTATTAAACGTTTCTGCACTGGCAAAGCGGCCGC

The endonuclease cleavage site of XhoI and NotI

The predicted combing site with miR-335-5p

The predicted miR-335-5p interaction with SIRT7-3'UTR

```
miR335-5p    3' ugUAAAAAGCAAU-AACGAGAACu 5'
                  |||||| |  | |||: ||||
SIRT7-3'UTR  5' acATTTTTAGCCATTTGTCCTTGa 3'
```

Supplementary Table 2.RNA Sequence mentioned in article

Name	Sequence (5'-3')
siRNA and miRNA transfection	
SIRT7-siRNA	CUGUGUUUGCUGAGGAUAAdTdT
control for SIRT7-siRNA	UUCUCCGAACGUGUCACGUTT
miR-335-5p mimics	UCAAGAGCAAUAACGAAAAAUGU
miR-335-5p inhibitor	ACAUUUUUCGUUAUUGCUCUUGA
control for miR-335-5p mimics	UUCUCCGAACGUGUCACGUTT
Primer in miR-335-5p expression assay	
miR-335-5p Reverse transcription primer	GTCGTATCCAGTGCAGGGTCCGAGGTATTCGCACTGGATACGACACATTTTT
miR-335-5p -F	TGCGCTCAAGAGCAATAACGAAA
Universal R primer	CCAGTGCAGGGTCCGAGGTATT
U6 -F	CGCTTCGGCAGCACATATAC
U6 -R	AAATATGGAACGCTTCACGA
